# An anti-sortilin affibody-peptide fusion inhibits sortilin-mediated progranulin degradation

**DOI:** 10.3389/fimmu.2024.1437886

**Published:** 2024-08-08

**Authors:** Moira Ek, Johan Nilvebrant, Per-Åke Nygren, Stefan Ståhl, Hanna Lindberg, John Löfblom

**Affiliations:** Department of Protein Science, School of Engineering Sciences in Chemistry, Biotechnology and Health, KTH Royal Institute of Technology, Stockholm, Sweden

**Keywords:** protein engineering, affibody molecule, sortilin (SORT1), progranulin (GRN), frontotemporal dementia (FTD), latozinemab, phage display

## Abstract

Heterozygous loss-of-function mutations in the *GRN* gene are a common cause of frontotemporal dementia. Such mutations lead to decreased plasma and cerebrospinal fluid levels of progranulin (PGRN), a neurotrophic factor with lysosomal functions. Sortilin is a negative regulator of extracellular PGRN levels and has shown promise as a therapeutic target for frontotemporal dementia, enabling increased extracellular PGRN levels through inhibition of sortilin-mediated PGRN degradation. Here we report the development of a high-affinity sortilin-binding affibody-peptide fusion construct capable of increasing extracellular PGRN levels *in vitro*. By genetic fusion of a sortilin-binding affibody generated through phage display and a peptide derived from the progranulin C-terminus, an affinity protein (A3-PGRN_C_15*) with 185-pM affinity for sortilin was obtained. Treating PGRN-secreting and sortilin-expressing human glioblastoma U-251 cells with the fusion protein increased extracellular PGRN levels up to 2.5-fold, with an EC_50_ value of 1.3 nM. Our results introduce A3-PGRN_C_15* as a promising new agent with therapeutic potential for the treatment of frontotemporal dementia. Furthermore, the work highlights means to increase binding affinity through synergistic contribution from two orthogonal polypeptide units.

## 1 Introduction

Frontotemporal dementia (FTD) is a form of non-Alzheimer’s dementia that is particularly common among cases of early-onset dementia (1). The disease is characterized by atrophy of the frontal and temporal lobes of the brain (2), leading to one of three main clinical phenotypes, with symptoms ranging from behavioral changes to language impairments (3). On a molecular level, FTD is characterized by aggregation of either microtubule-associated protein tau (MAPT), TAR DNA-binding protein 43 (TDP-43), or fused-in-sarcoma (FUS) (3).

The disease has a strong genetic component, and a family history of FTD is present in up to 40% of all cases (4–6). The majority of these cases are due to mutations in the genes encoding either microtubule-associated protein tau (*MAPT*), progranulin (*GRN*), or chromosome 9 open reading frame 72 (*C9orf72*) (7). Heterozygous mutations in the *GRN* gene, encoding the protein progranulin (PGRN), are present in about 5-10% of all FTD cases, and up to 26% of familial FTD cases (4, 8–11). To date, at least 130 different disease-associated mutations have been identified in the *GRN* gene (7).

PGRN is a 593-amino acid glycoprotein consisting of seven and a half cysteine-rich granulin domains (12–14), into which the protein can be cleaved by both intra- and extracellular proteases (15, 16). PGRN and the different granulins exert a multitude of, sometimes opposing (16), functions, including roles in lysosomal function (17, 18) and as a neurotrophic factor (19–21). The identified pathogenic mutations in the *GRN* gene are believed to cause FTD through haploinsufficiency (9, 22), as they are associated with more than 50% decreased PGRN levels in plasma and CSF of mutation carriers compared to controls (19, 23–25). Thus, increasing PGRN levels to the normal range is currently investigated as a potential therapeutic strategy for the treatment of FTD with *GRN* mutations (FTD-*GRN*).

An interesting target to this end is the PGRN clearance receptor, sortilin. This is a type I membrane protein, with the main luminal domain forming a 10-bladed beta propeller with an inner tunnel into which both PGRN and the neuropeptide neurotensin (NT) bind (26–29). PGRN interacts with sortilin through the PGRN C-terminal tail, leading to endocytosis and lysosomal localization of PGRN (28, 29). Thus, sortilin is a negative regulator of extracellular PGRN levels (28, 30). Importantly, the neurotrophic effects of PGRN have been shown to be independent of sortilin binding (20, 21), making sortilin an attractive target for efforts to increase extracellular PGRN levels. Blocking the PGRN-sortilin interaction has been demonstrated to increase PGRN levels *in vitro* and *in vivo* in several studies (31–33). Most notably, the anti-sortilin IgG1 latozinemab (32) is currently in phase 3 clinical trials for FTD-*GRN* (clinicaltrials.gov ID: NCT04374136), establishing sortilin targeting as a promising treatment strategy for FTD-*GRN*.

**Figure 1:**
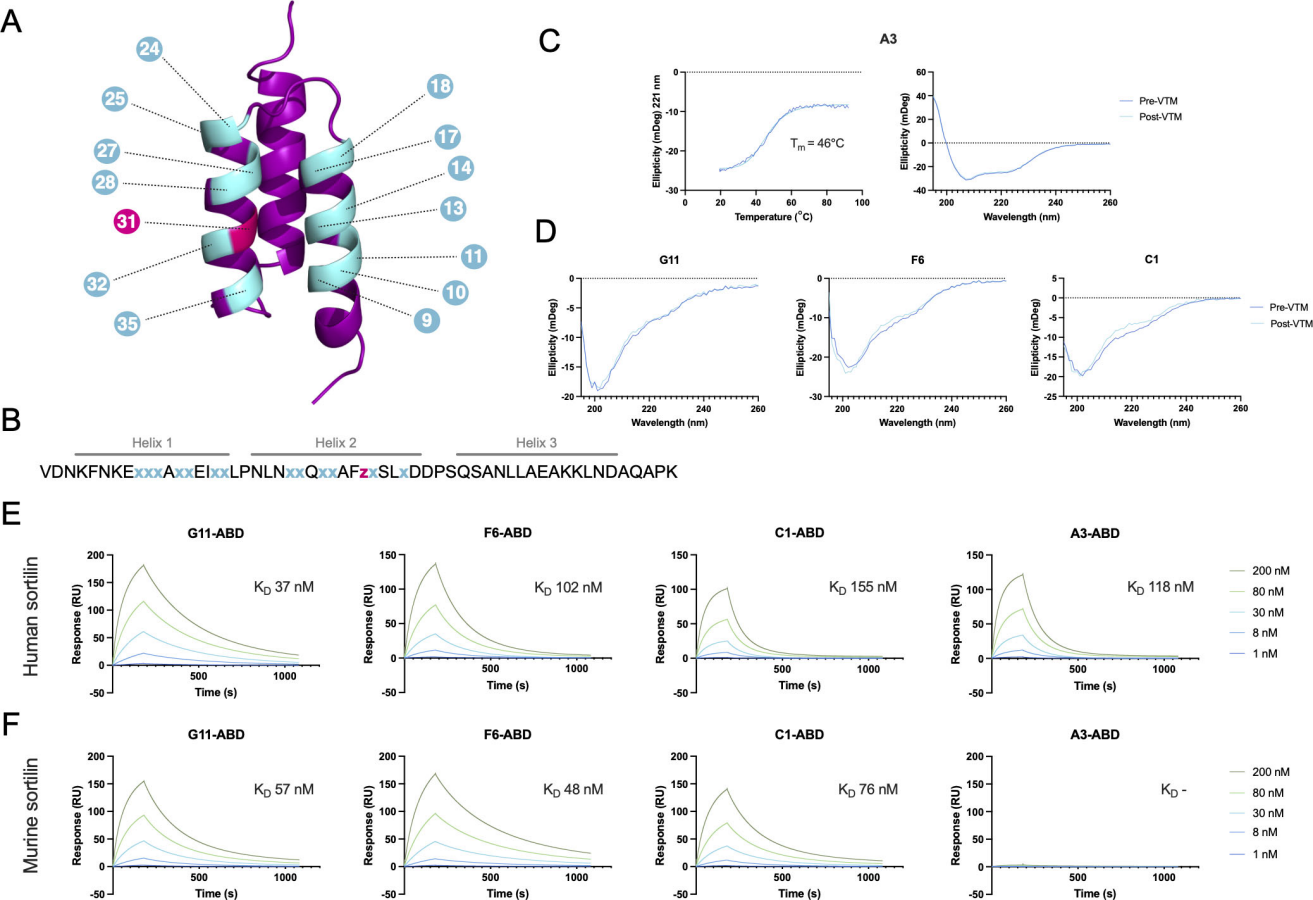
Schematic overview of the affibody scaffold and initial characterization of anti-sortilin affibodies. **(A, B)** Schematic of a three-helical affibody molecule **(A)** and the sequence of the affibody scaffold **(B)**, highlighting the 14 randomized positions in the phage display library. Gray lines over the sequence indicate the expected positions of the three alpha helices. Randomized positions are shown in cyan (x), indicating an equal mixture of all amino acids excluding Cys and Pro, or pink (z), indicating a distribution with 60% Ile and 10% each of His, Tyr, Lys, and Asp. **(C)** Circular dichroism spectroscopy of the anti-sortilin affibody variant A3, including variable temperature measurements (VTM) at 221 nm (left), and secondary structure determination before and after thermal melting (right). VTM measurements are shown in dark blue, with the 4-parameter curve fit from which the melting temperature was estimated shown in turquoise. **(D)** Circular dichroism spectroscopy of the anti-sortilin affibody variants G11, F6 and C1, showing secondary structure determination before and after thermal melting. **(E, F)** Surface plasmon resonance (SPR) sensorgrams showing the interaction between the anti-sortilin affibodies and human **(E)** or murine **(F)** sortilin. Affibody-ABD fusion proteins were captured on a sensor chip surface functionalized with HSA, followed by injection of sortilin, as indicated in the figure. Inserts indicate the equilibrium dissociation constant for the interaction between the respective affibody and sortilin, as estimated by a 1:1 Langmuir model fit.

As an alternative to antibodies, targeted therapies can also be based on smaller engineered antibody domains or non-Ig-derived alternative scaffold proteins (34), possessing several potential advantages over antibodies in cases where Fc-mediated effector functions are undesirable. One such class of alternative scaffold proteins is affibody molecules. These are small (58-amino acid, ~6.5 kDa) three-helical affinity proteins based on the Z domain, a derivative of *Staphylococcus aureus* protein A (35, 36). Typically, 13 or 14 surface-exposed positions in helix 1 and 2 are randomized to generate a library from which affibody molecules with affinity for new targets can be selected (37) (**Figure 1A**). In comparison to antibodies, the affibody scaffold notably lacks inherent effector functions, as well as disulfide bonds, and generally benefits from high stability. Its small size also makes it amenable to production in bacterial hosts or through chemical synthesis. The small size has furthermore prompted modular approaches, such as tandem fusions of several affibody molecules to increase target affinity (38), fusion of affibody molecules to enzymes (39, 40) or anti-idiotypic affibody molecules (41, 42) in prodrug approaches, or fusion with albumin-binding domains (ABD) for half-life extension *in vivo* (43). An IL-17A-targeting, ABD-containing affibody construct is currently being investigated in phase 3 clinical trials (NCT05623345, NCT05905783), having demonstrated good safety and tolerability in humans (44).

Here, we introduce a novel affibody-based sortilin-targeting approach for increasing extracellular PGRN levels. First, sortilin-specific affibody molecules were selected using phage display technology. We next explored genetic fusions of several affibody candidates to short peptides derived from the sortilin-binding C-terminal part of PGRN, to possibly increase affinity via synergistic binding. The most optimal fusion construct resulted in a dramatic increase (>380-fold) in binding affinity to sortilin and was shown to increase extracellular PGRN levels *in vitro* in sortilin-expressing, PGRN-secreting human glioblastoma U-251 cells, with an EC_50_ comparable to that of latozinemab, suggesting a therapeutic potential. Further, this is the first example of a modular approach where fusion of an affibody molecule to a natural peptide targeting the same receptor leads to greatly improved affinity and biological activity.

## 2 Materials and methods

### 2.1 Labeling of targets and secondary reagents

Murine (R&D Systems, 2934-ST) and human (R&D Systems, 3154-ST) sortilin proteins were biotinylated for 1 h using EZ-Link Sulfo-NHS-LC-Biotin (Thermo Fisher Scientific) in a 25-fold molar excess followed by dialysis to PBS using a Slide-A-lyzer 10K MWCO (Thermo Fisher Scientific). Human serum albumin (HSA, 20 mg/ml) was labeled with Alexa Fluor™ 647 succinimidyl ester (Invitrogen A-20006, 0.5 mg/ml) in carbonate buffer (0.1 M, pH 8.5) for 3 hours at room temperature, followed by quenching with a 1000-fold molar excess of glycine. Labeled HSA was purified by gel filtration into PBS (pH 7.4) using PD-10 columns (Cytiva), according to the manufacturer’s instructions. Concentration of the proteins was determined using absorbance at 280 nm.

### 2.2 Phage display selection of anti-sortilin affibody molecules

Phage display selections of affibodies binding to sortilin were performed essentially as described by Giang et al. (45), using a previously described combinatorial phage library of the Z domain with randomization in 14 positions (46) (**Figures 1A, B**). Briefly, four cycles of bio-panning were conducted at room temperature in PBSTB (PBS with 0.1% Tween20 and 3% *w/v* BSA) with decreasing target concentration and increasing washing in each round. In the first two cycles, biotinylated sortilin was pre-immobilized onto paramagnetic streptavidin beads (M-280, Thermo Fisher Scientific) and in the later cycles, phages were incubated with biotinylated target in solution before capture of target-phage complexes on beads. Parallel selections were performed using either murine or human sortilin as target, and bound phages were eluted by either trypsin cleavage (Gibco by Life Technologies, #15090-046, 2.5 mg/ml) or acid (0.3 M HAc, pH 2.8). Starting in the third round, two additional selection tracks were initiated from pooled phage stocks from the two elution strategies for each target (murine/human sortilin) and subjected to competitive elution (1 h incubation) using 5 µM neurotensin (Sigma, N6383) in PBST (PBS supplemented with 0.05% Tween20, pH 7.4). 47 random clones from each of the six selection tracks were assayed by phage-ELISA for binding to human and murine sortilin (immobilized in separate wells at 2.5 µg/ml in 0.1 M carbonate buffer pH 9.6). Binding signal from an albumin-binding domain (ABD_wt_) tag fused to the C-terminus of phage p3-displayed affibody to immobilized HSA was used to normalize binding signals in ELISA (45). Clones with positive ELISA-signals were sent for sequencing.

### 2.3 Production and purification of recombinant proteins

Constructs of the format His_6_-Z (Z) were cloned into a pT7 vector. First- and second-generation construct genes containing an ABD035 (47) (**Supplementary Table S1**) were synthesized as gene fragments and cloned into a pET21 expression vector (Twist Bioscience, South San Francisco, CA, USA) with an N-terminal hexahistidine tag for immobilized metal affinity chromatography (IMAC) purification. Constructs were of the formats His_6_-Z-G_4_S-ABD035 (Z-ABD), His_6_-Z-G_4_S-ABD035-PGRN_C_21* (Z-ABD-PGRN_C_21*), His_6_-ABD035-G_4_S-Z-PGRN_C_21* (ABD-Z-PGRN_C_21*), His_6_-ABD035-PGRN_C_21* (ABD-PGRN_C_21*), His_6_-ABD035-G_4_S-A3-PGRN_C_X* (ABD-A3-PGRN_C_X*), His_6_-ABD035-G_4_S-A3-(G_4_S)_3_-PGRN_C_X* (ABD-A3-(G_4_S)_3_-PGRN_C_X*), and His_6_-ABD035-G_4_S-A3-(G_4_S)_3_-NT_C_3 (ABD-A3-(G_4_S)_3_-NT_c_3). Plasmids were transformed into *Escherichia coli* BL21 Star cells (Thermo Fisher Scientific) using a standard heat shock transformation protocol, followed by protein production and purification. Briefly, cells were cultivated in tryptic soy broth with yeast extract (TSB+Y, Merck) supplemented with 100 μg/ml of carbenicillin or 50 μg/ml of kanamycin at 37°C and 150 rpm shaking. At an OD_600_ of approximately 0.7, protein expression was induced with isopropyl β-D-1-thiogalactopyranoside (IPTG) to a final concentration of 1 mM. The cultures were incubated at 25°C and 150 rpm for approximately 16 hours prior to harvest. Cells were lysed by sonication, and proteins were purified by native IMAC on HisPur Cobalt Resin (Thermo Fisher Scientific) at 4°C. Purified proteins were buffer-exchanged to PBS (pH 7.4) using PD-10 columns (Cytiva), according to the manufacturer’s recommendations, and analyzed by SDS-PAGE (NuPAGE, Invitrogen), bicinchoninic acid assay (Pierce, Thermo Fisher Scientific) and mass spectrometry (MS, 4800 MALDI TOF/TOF, Applied Biosystems/MDS SCIEX). Z-ABD format proteins were analyzed by size exclusion chromatography (Superdex 75 Increase 5/150 GL, Cytiva).

### 2.4 Surface plasmon resonance for affibody screening and affinity determination

Target binding was assessed by surface plasmon resonance (SPR) using Biacore 3000 (screening of affibody clones after phage display selections), Biacore T200 (screening of first-generation affibody-PGRN fusion constructs), and Biacore 8K (affinity determination of second-generation affibody-PGRN fusion constructs) instruments (Cytiva, Uppsala, Sweden), respectively. In all cases, HSA was immobilized through amine coupling on a CM5 sensor chip according to the manufacturer’s recommendations, using 10 mM sodium acetate pH 4.5 as the immobilization buffer, with a reference surface being only activated and inactivated. PBST was used as the running buffer in all binding experiments. 30-100 RU of ABD035-containing constructs were captured on the HSA surface, followed by injection of 1-200 nM human (R&D Systems 3154-ST) or murine (R&D Systems 2934-ST) sortilin for 180 s at 30 µl/min and 25°C. Dissociation was recorded for 15 min (screening experiments) or 60 min (affinity determination experiments) prior to regeneration with 10 mM HCl for 30 s at 30 µl/min. Kinetic constants were estimated using 1:1 Langmuir curve fits of sensorgrams from which a reference capture of the respective ABD035-containing construct with a 0 nM sortilin injection had been subtracted.

### 2.5 Circular dichroism spectroscopy for secondary structure and melting temperature determination

Circular dichroism (CD) spectroscopy was performed on affibody molecules in a His_6_-Z format to verify the secondary structure content, using a Chirascan Circular Dichroism Spectrometer (Applied Biophysics Ltd, Leatherhead, UK). All analyses were performed at a concentration of 0.1-0.2 mg/ml in PBS (pH 7.4) and a 1 mm path length. Secondary structure content was assessed by measuring ellipticity at 20°C from 195 nm to 260 nm. For alpha helical molecules, the thermal stability was evaluated by measuring the change in ellipticity at 221 nm when heating from 20°C to 95°C at 5°C/min. After cooling to 20°C, another spectrum was recorded (195-260 nm) to assess refolding capacity. The melting temperature (T_m_) was obtained as the inflection point of a 4-parameter fit of the variable temperature measurement data in Prism version 10 (GraphPad, Boston, MA, USA).

### 2.6 Mammalian cell cultivation

U-251 (JCRB IFO50288) human glioblastoma cells were cultivated in Minimum Essential Medium with glutamine (Gibco MEM, Thermo Fisher Scientific 31095) supplemented with 10% fetal bovine serum (FBS, Gibco, Fisher Scientific). PC-3 (ATCC CRL-1435) human prostatic adenocarcinoma cells were cultivated in Roswell Park Memorial Institute 1640 medium with glutamine (Gibco RPMI 1640, Thermo Fisher Scientific 21875) supplemented with 10% FBS. Cells were grown at 37°C in a 5% CO_2_ atmosphere and were detached from culture flasks using TrypLE™ EXPRESS (Thermo Fisher Scientific 12605), according to the supplier’s recommendations.

### 2.7 Flow cytometric analysis of sortilin binding on U-251 and PC-3 cells

Primary constructs were pre-incubated with secondary constructs in PBS+1% BSA on ice for a minimum of 1 hour prior to their addition to cells. ABD035-containing constructs (100 nM) were pre-incubated with HSA-Alexa Fluor 647 (200 nM), and a positive control Human Sortilin Antibody (R&D systems MAB31541, 0.625 μg/ml) was pre-incubated with Alexa Fluor 647 Goat Anti-Mouse Antibody (Invitrogen A21235, 2.86 μg/ml). 2×10^5^ U-251 or PC-3 cells per sample were washed in 200 μl ice-cold PBS+1% BSA, followed by incubation with 200 μl pre-incubated affibody construct or control antibody for 40 min at 4°C. Cells were washed twice, followed by resuspension in 200 μl ice-cold PBS+1% BSA for analysis on a Cytoflex S flow cytometer (Beckman Coulter, Brea, CA, USA), gating single cells based on forward/side scatter, and using a 638 nm laser for fluorophore excitation and a 660/10 BP filter for detection.

### 2.8 PGRN clearance assay

A PGRN clearance assay was performed essentially as described by Miyakawa et al. (31). Briefly, 1×10^4^ U-251 cells in 100 μl of the appropriate medium were seeded per well of a 96-well plate (Nunclon™ Delta Surface, Thermo Fisher Scientific). The cells were incubated for 24 h prior to the addition of fresh medium containing different concentrations of protein constructs, in triplicates. After 72 h, supernatants were collected, and PGRN concentrations were quantified using Human Progranulin DuoSet ELISA (R&D Systems DY2420), according to the manufacturer’s instructions. Absorbance was measured at 450 and 540 nm using a ClarioStar (BMG Labtech, Ortenberg, Germany) plate reader, and PGRN levels were normalized against untreated cells to obtain the PGRN level fold change upon treatment. EC_50_ values were obtained from a 4-parameter fit in Prism version 10 (GraphPad, Boston, MA, USA). Means and standard deviations were calculated from the EC_50_ values from N=3 independent experiments. In addition to in-house-produced affibodies and affibody-peptide fusion proteins, a latozinemab biosimilar (ProteoGenix PX-TA1676) was evaluated. The statistical significance of the difference in EC_50_ values between ABD-A3-PGRN_C_15* and latozinemab was evaluated using a paired two-tailed t-test in Prism version 10.

## 3 Results

### 3.1 Isolation of sortilin-binding affibody molecules through phage display

In order to obtain sortilin-binding affibody molecules, phage display selections were performed against human and murine sortilin. A previously described (45) M13 filamentous phage library of 3×10^10^ affibody variants with randomizations in 14 positions (**Figures 1A, B**) was subjected to four rounds of panning against decreasing concentrations of either human or murine sortilin. Target-binding clones were identified by phage ELISA-screening of a total of 282 randomly selected clones. DNA sequencing of 54 target-binding clones showed 31 unique sequences. Among these, 12 clones representing major sequence clusters were chosen for subcloning to a His_6_-Z-ABD_wt_ format, expressed in *E. coli*, and purified by IMAC. After screening by surface plasmon resonance (SPR, data not shown), clones G11 (mSort track), F6 (mSort track), C1 (mSort track), and A3 (hSort track) were chosen for further characterization.

### 3.2 Production and characterization of sortilin-binding affibody molecules

The anti-sortilin affibody clones G11, F6, C1 and A3 were produced in soluble format in *E. coli*, in Z-ABD and Z formats. Following IMAC purification, >95% affibody monomeric state was confirmed by size exclusion chromatography of Z-ABD format proteins (**Supplementary Figure S1**), and the secondary structure and thermal stability of the affibody molecules was investigated using circular dichroism spectroscopy of Z format proteins. Interestingly, only variant A3 displayed an expected alpha helical structure content. Variable temperature measurements of A3 showed a melting temperature (T_m_) of 46°C, and complete refolding after heat treatment (**Figure 1C**). Variants G11, F6, and C1 showed CD spectra largely consistent with a random coil conformation (**Figure 1D**).

The affinity of the affibody molecules to human and murine sortilin was assessed by SPR, by capturing Z-ABD constructs on a sensor chip immobilized with HSA, followed by injection of five concentrations (ranging from 1 nM to 200 nM) of human and murine sortilin, respectively. No sortilin binding was observed to the negative control Z_wt_-ABD construct, confirming the absence of potential interaction between sortilin and the ABD or the affibody scaffold. Interestingly, the three affibody candidates selected against murine sortilin (G11, F6 and C1) showed binding to both human and murine sortilin, whereas clone A3 only displayed binding to human sortilin (**Figures 1E, F**). The equilibrium dissociation constants (K_D_) were in the range of 37 to 155 nM and 48 to 76 nM for human and murine sortilin, respectively.

### 3.3 Design of first-generation affibody-PGRN fusion constructs

**Figure 2:**
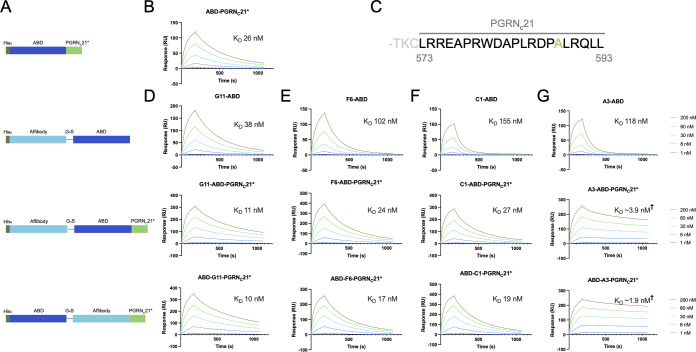
SPR screening of first-generation affibody-PGRN_C_ fusion proteins. **(A)** Schematic overview of the evaluated constructs. In addition to Z-ABD-PGRN_C_21* and ABD-Z-PGRN_C_21* format constructs, Z-ABD and ABD-PGRN_C_21* constructs were included as controls. **(B)** SPR sensorgram showing the interaction between the PGRN C-terminus, captured on an HSA-coated sensor chip, and human sortilin. **(C)** Sequence of the PGRN C-terminus, with the peptide denoted PGRN_C_21 highlighted. Numbering indicates amino acid numbers from the start codon. Ala588 is shown in green, indicating the position of the A588G mutation in the PGRN_C_21* peptide. **(D-G)** SPR sensorgrams showing the interaction between Z-ABD, Z-ABD-PGRNC21*, and ABD-Z-PGRN_C_21* format constructs and human sortilin, for affibodies G11 **(D)**, F6 **(E)**, C1 **(F)**, and A3 **(G)**, respectively. Due to a low degree of dissociation within the measured 15-minute dissociation time period precluding accurate estimation of kinetic constants, values marked ^†^ are low-accuracy approximations.

The sortilin-progranulin interaction is known to be mediated by the C-terminal tail of PGRN (28, 29), and previous studies have reported that the three last amino acids in the C-terminus (QLL) are essential for binding to sortilin (29). Thus, it was hypothesized that a peptide derived from the PGRN C-terminus might be used as an extension to a sortilin-binding affibody to obtain a biparatopic sortilin-binding fusion protein. To test this hypothesis, fusion proteins of the four sortilin-binding affibodies and the human PGRN C-terminus were created (**Figure 2A**).

Previous work has demonstrated that the 24 last amino acids of PGRN (T570-L593, here denoted PGRN_C_24) are sufficient for full binding to sortilin, whereas shorter versions PGRN_C_9 (R585-L593) and PGRN_C_6 (A588-L593) were found to interact with sortilin to a lesser extent (29). As amino acid 22 from the PGRN C-terminus is a cysteine (C572), PGRN_C_21 (L573-L593) was selected for the initial affibody-PGRN fusion proteins in order to avoid disulfide bond formation (**Figure 2C**). In addition, others have shown that an A588G mutation in PGRN increases proteolytic stability by disruption of a neutrophil elastase cleavage site, with no effect on PGRN uptake by cells (33). The A588G mutation was confirmed to not affect sortilin binding by SPR (data not shown), and the PGRN_C_21 peptide carrying the A588G mutation (henceforth PGRN_C_21*) was chosen as the initial PGRN peptide moiety for fusion. Given that a free PGRN C-terminus has been shown to be required for sortilin binding (21, 29) and that the binding epitopes of the affibodies on sortilin were not known, we designed a set of eight fusion constructs with the ABD moiety located either N-terminally or between the affibody and PGRN peptide, to serve as a spacer (**Figure 2A**).

### 3.4 Evaluation of first-generation affibody-PGRN fusion constructs

Constructs of the format Z-ABD, Z-ABD-PGRN_C_21* and ABD-Z-PGRN_C_21*for each of the four sortilin-binding affibodies (Z), as well as ABD-PGRN_C_21* were expressed in *E. coli* and purified by IMAC. Potential simultaneous binding to human sortilin by the affibody and PGRN_C_21* moieties was investigated by SPR, as described above. Fusion of affibodies G11, C1 and F6 to PGRN_C_21* led to limited or no improvements in apparent affinity compared to the parental peptide or affibodies (**Figures 2B, D–F**), indicating that the affibody and peptide epitopes on sortilin were suboptimal for simultaneous binding. In contrast, fusion of the PGRN_C_21* peptide to anti-sortilin affibody A3 led to a dramatic decrease in dissociation rate (**Figure 2G**), indicating avidity and hence compatibility between the A3 and PGRN_C_21* epitopes for simultaneous binding. Notably, the dissociation rate of the A3-PGRN_C_21* constructs was too slow to enable accurate determination of kinetic constants within the tested 15-minute dissociation period. Qualitatively, the position of the ABD in the A3-PGRN_C_21* constructs seemed to have no major effect on binding, indicating that the ABD moiety was not required as a spacer between A3 and PGRN_C_21*. This raised the question of whether parts of the PGRN_C_21* peptide also merely acted as a spacer, rather than directly contributing to the co-operative binding.

### 3.5 Optimization of an affibody-PGRN fusion construct

**Figure 3:**
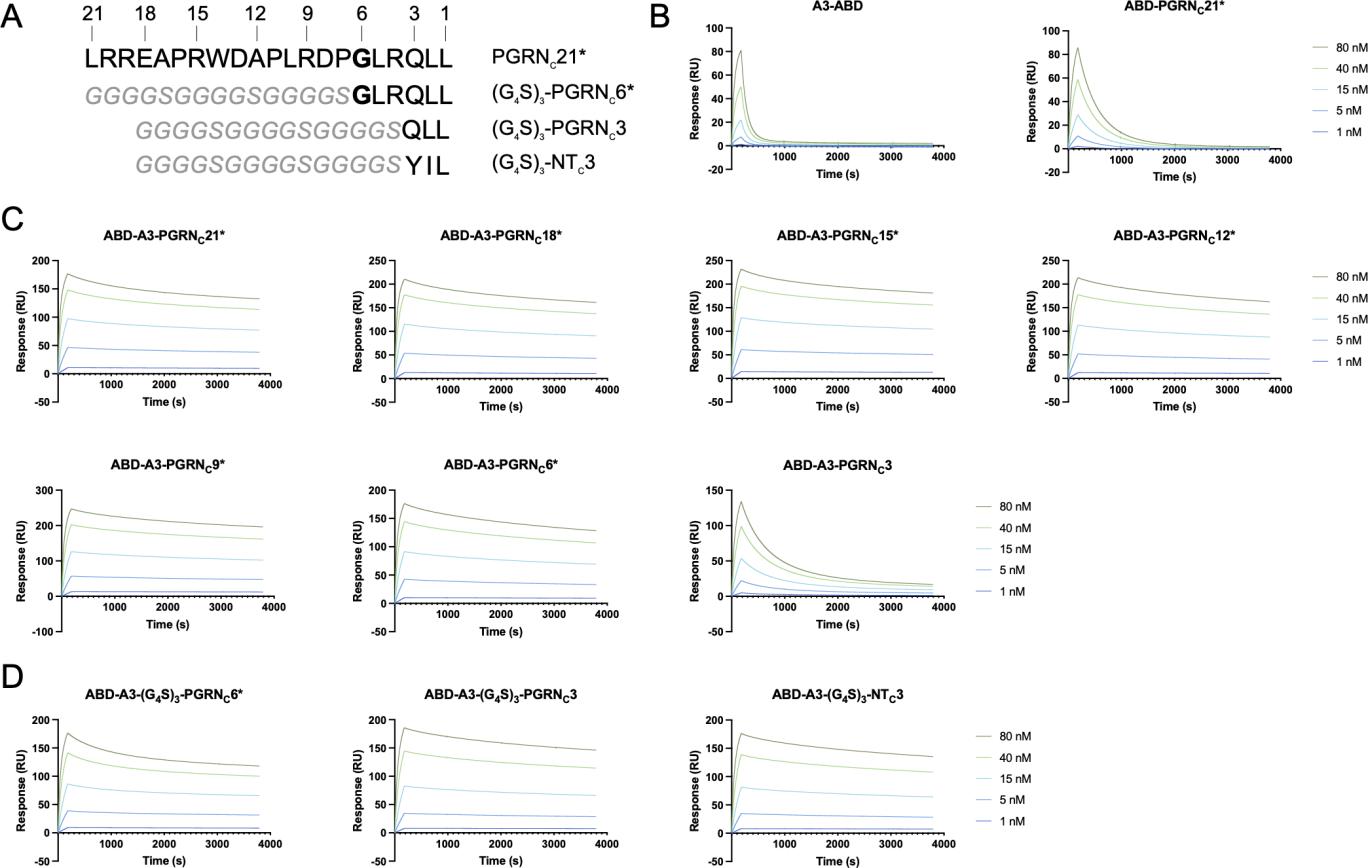
SPR-based affinity determination of second-generation A3-PGRN_C_ fusion proteins. **(A)** Sequence overview of the various PGRN_C_*, (G_4_S)_3_-PGRN_C_*, and (G_4_S)_3_-NT_C_3 peptides. Numbers indicate the peptide length, as counted from the C-terminus. The site of the PGRN A588G mutation is indicated in bold. **(B-D)** Representative sensorgrams showing the interaction of the individual affibody and peptide moieties **(B)**, second-generation affibody-peptide fusion constructs **(C)**, and affibody-peptide fusions with a flexible linker **(D)** with human sortilin. The length of the peptide moieties, according to **(A)**, are indicated in the subfigure headings. ABD-containing constructs were captured on an HSA-coated sensor chip, followed by sortilin injection, and reference subtraction. Displayed sensorgrams are representative examples of n=3 technical replicates.

To investigate whether all 21 amino acids of the PGRN_C_21* peptide are required for simultaneous sortilin binding in the A3-PGRN_C_ fusion protein, constructs of the format ABD-A3-PGRN_C_X*, where X={3, 6, 9, 12, 15, 18, 21} (**Figure 3A**), were produced and purified. The affinities for human sortilin were evaluated by SPR, as described above. Notably, a dramatic difference in dissociation rate and K_D_ (>13-fold) was seen between the ABD-A3-PGRN_C_3 (K_D_=3.9 nM) and the ABD-A3-PGRN_C_6* (K_D_=289 pM) constructs (**Figures 3B, C**, **Table 1**), indicating that PGRN_C_6* is sufficient to both bind to sortilin and serve as a spacer between the A3 and PGRN_C_ epitopes. Moreover, a somewhat longer peptide moiety resulted in an even stronger interaction. All tested ABD-A3-PGRN_C_X* fusion proteins with a PGRN moiety of 9 or more amino acids had smaller K_D_ values than ABD-A3-PGRN_C_6*, with a minimum K_D_ value of 185 pM for ABD-A3-PGRN_C_15* (**Figure 3C**, **Table 1**).

**Table 1:** Summary of kinetic constants for the interaction of A3-PGRN_C_ fusion proteins and controls with human sortilin, as determined by SPR.

Construct	Apparent k_on_ (1/Ms)	Apparent k_off_ (1/s)	Apparent K_D_ (nM)
A3-ABD	1.16 × 10^5^ ± 4.64 × 10^3^	8.11 × 10^-3^ ± 1.10 × 10^-4^	70.4 ± 3.69
ABD-PGRN_C_21*	2.12 × 10^5^ ± 6.94 ×10^3^	3.37 × 10^-3^ ± 5.56 × 10^-5^	15.9 ± 0.22
ABD-A3-PGRN_C_3	7.52 × 10^5^ ± 1.84 × 10^4^	2.95 × 10^-3^ ± 1.41 × 10^-5^	3.93 ± 0.12
ABD-A3-PGRN_C_6*	2.81 × 10^5^ ± 4.71 × 10^2^	8.09 × 10^-5^ ± 1.31 × 10^-6^	0.289 ± 0.0041
ABD-A3-PGRN_C_9*	2.65 × 10^5^ ± 4.71 × 10^2^	5.95 × 10^-5^ ± 9.63 × 10^-7^	0.224 ± 0.0029
ABD-A3-PGRN_C_12*	2.83 × 10^5^ ± 4.71 × 10^2^	7.12 × 10^-5^ ± 1.07 × 10^-6^	0.251 ± 0.0036
ABD-A3-PGRN_C_15*	3.21 × 10^5^ ± 2.45 × 10^3^	5.94 × 10^-5^ ± 8.52 × 10^-7^	0.185 ± 0.0016
ABD-A3-PGRN_C_18*	3.08 × 10^5^ ± 1.41 × 10^3^	6.69 × 10^-5^ ± 8.34 × 10^-7^	0.217 ± 0.0021
ABD-A3-PGRN_C_21*	3.25 × 10^5^ ± 1.70 × 10^3^	6.98 × 10^-5^ ± 1.12 × 10^-6^	0.215 ± 0.0022
ABD-A3-(G_4_S)_3_-PGRN_C_3	2.05 × 10^5^ ± 5.0 × 10^2^	6.34 × 10^-5^ ± 7.54 × 10^-7^	0.310 ± 0.0038
ABD-A3-(G_4_S)_3_-PGRN_C_6*	2.88 × 10^5^ ± 1.63 × 10^3^	9.16 × 10^-5^ ± 4.55 × 10^-7^	0.319 ± 0.0033
ABD-A3-(G_4_S)_3_-NT_C_3	2.21 × 10^5^ ± 8.16 × 10^2^	6.70 × 10^-5^ ± 8.34 × 10^-7^	0.303 ± 0.0047

Values for k_on_, k_off_ and K_D_ were obtained from a 1:1 Langmuir curve fit of reference subtracted sensorgrams. Values are given as mean±SD from n=3 technical replicates. * indicates the presence of the A588G mutation in PGRN-derived peptides.

To investigate whether this increase in apparent affinity in A3-PGRN_C_ constructs with longer PGRN_C_ peptides was due to the N-terminal amino acids of the PGRN_C_ moiety interacting with sortilin or merely serving as a spacer, the corresponding amino acids were in additional constructs replaced with a flexible linker. Constructs of the format ABD-A3-(G_4_S)_3_-PGRN_C_X*, where X={3, 6}, were tested for binding to human sortilin in SPR. In contrast to ABD-A3-PGRN_C_3 (K_D_=3.9 nM), which provided only a modest decrease in K_D_ versus affibody alone, ABD-A3-(G_4_S)_3_-PGRN_C_3 (K_D_=310 pM) had a K_D_ value in the same range as the fusion proteins with longer PGRN moieties, such as ABD-A3-PGRN_C_6* (K_D_=289 pM). This indicates that PGRN_C_3 is sufficient to convey sortilin binding, when spaced appropriately from A3. The very similar apparent K_D_ values of the ABD-A3-(G_4_S)_3_-PGRN_C_3 (310 pM) and ABD-A3-(G_4_S)_3_-PGRN_C_6* (319 pM) constructs furthermore indicate that the 3 N-terminal-most amino acids of the PGRN_C_6* peptide (GLR) mainly serve as a spacer in the ABD-A3-PGRN_C_6* construct, rather than being essential for sortilin binding (**Figure 3D**, **Table 1**). However, the higher affinities of ABD-A3-PGRN_C_18* (217 pM) and ABD-A3-PGRN_C_21* (215 pM) compared to ABD-A3-(G_4_S)_3_-PGRN_C_3 (310 pM) and ABD-A3-(G_4_S)_3_-PGRN_C_6* (319 pM), respectively, indicate that the N-terminal portion of the longer PGRN_C_ peptides might in fact contribute somewhat to binding in addition to serving as a spacer.

Additionally, an initial comparison between the 3 C-terminal-most amino acids of the sortilin-binding peptide neurotensin (YIL), known to bind to the same epitope as PGRN (27, 28, 48), and those of PGRN (QLL) was also made, in the form of the construct ABD-A3-(G_4_S)_3_-NT_C_3. The ABD-A3-(G_4_S)_3_-NT_C_3 construct (K_D_=303 pM) displayed a similar affinity for sortilin as compared to ABD-A3-(G_4_S)_3_-PGRN_C_3 (K_D_=310 pM, **Figure 3D**, **Table 1**), suggesting that the QLL and YIL extensions contribute to the sortilin binding with equal potencies.

### 3.6 Binding to sortilin on cells

**Figure 4:**
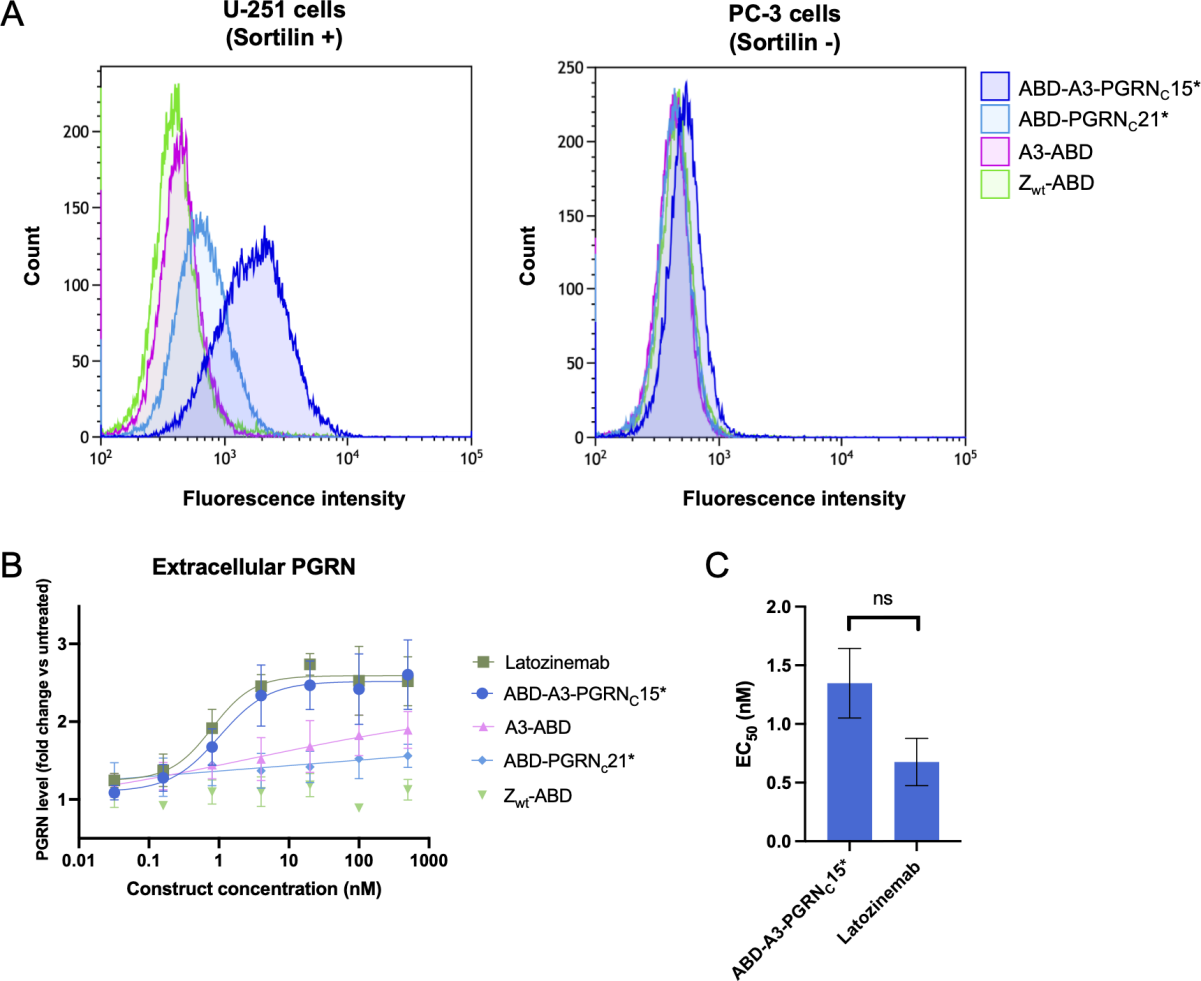
Characterization of cell binding and *in vitro* biological activity of affibody-PGRN fusion proteins. **(A)** Characterization of sortilin binding on cancer cells by flow cytometry. Binding of anti-sortilin affibody A3, the PGRN C-terminus, and A3-PGRN_C_* constructs to sortilin-expressing U-251 cells (left) and the control cell line PC-3 (right) was evaluated in comparison to the non-binding affibody control Z_wt_. 100 nM of ABD-fusion proteins were pre-incubated with HSA-Alexa Fluor 647 prior to addition to the cells, enabling fluorescent detection of binding. ABD-A3-PGRN_C_15* is shown as a representative example of the high-affinity A3-PGRN_C_* fusion proteins. Data for all constructs is summarized in **Supplementary Figure S3**. **(B, C)** Characterization of the biological activity of sortilin-binding constructs. **(B)** Extracellular PGRN levels as measured by ELISA after 72 h incubation of sortilin-expressing, PGRN-secreting U-251 cells with increasing concentrations of A3-PGRN fusion constructs or controls. Latozinemab concentrations indicate paratope (two per IgG) concentrations. PGRN levels are reported as fold change versus untreated cells. Displayed data is a representative example of N=3 independent experiments, with n=3 biological replicates in each. For each construct except Z_wt_, a 4-parameter curve fit to the data is shown. **(C)** Summary of PGRN clearance EC_50_ values for an affibody-PGRN fusion protein and a latozinemab biosimilar. EC_50_ values were calculated as the inflection point of a 4-parameter curve fit to the PGRN fold change *vs*. construct concentration curve, and are reported as mean ± SD from N=3 independent experiments with n=3 biological replicates in each. Control constructs A3-ABD, ABD-PGRN_C_21* and Z_wt_-ABD failed to reach a plateau within the tested concentration range, prohibiting EC_50_ determination. ns, not significant.

Next, the constructs’ binding capabilities to sortilin in the more biologically relevant context of the cell surface was investigated. The binding of i) anti-sortilin affibodies, ii) the PGRN C-terminus, iii) A3-PGRN_C_ fusion proteins, and iv) a negative control affibody (Z_wt_) to the sortilin high-expressing glioblastoma cell line U-251 and the control prostate cancer cell line PC-3 was compared. Cell line sortilin expression status was verified using a positive control antibody (**Supplementary Figure S2**). To ensure simultaneous binding to sortilin and HSA, ABD-containing constructs were pre-incubated with an excess of fluorophore-labelled human serum albumin (HSA-Alexa Fluor 647) prior to addition to cells and analysis by flow cytometry. All constructs showed binding to U-251 cells (**Figure 4A**, **Supplementary Figure S3**), except the negative control affibody Z_wt_, which did not show any binding to U-251 or PC-3 cells (**Supplementary Figure S2**). Notably, the constructs with highest affinities for sortilin also showed a small fluorescence shift relative negative control affibody for the negative control cell line PC-3 (**Figure 4A**). This is in line with reports of some low sortilin expression on PC-3 cells, as determined by western blot (49), and the high affinity of these constructs for sortilin.

### 3.7 Effect on extracellular PGRN levels *in vitro*

The biological activity of affibody-PGRN_C_ peptide fusions was evaluated in a cell-based progranulin clearance assay. U-251 cells both express sortilin and secrete progranulin, making them a suitable model system to study the effect of sortilin binders on extracellular PGRN levels. The ABD-A3-PGRN_C_15* construct, showing the highest affinity for sortilin in SPR, was chosen as the lead affibody-peptide fusion candidate, and evaluated in comparison to i) A3 alone, ii) the PGRN C-terminus alone, iii) an affibody negative control (Z_wt_), and iv) latozinemab. Under the employed experimental conditions, supernatant PGRN levels in untreated cells, constituting the baseline, were 1.52±0.15 ng/ml. At the highest tested concentration (500 nM), A3 and PGRN_C_ alone did not reach more than a 1.48±0.31- and 1.44±0.27-fold increase in extracellular PGRN levels compared to untreated cells, respectively, with the lack of a plateau making EC_50_ determination unreliable. In contrast, ABD-A3-PGRN_C_15* increased extracellular PGRN levels 2.50±0.19-fold compared to untreated cells, with an EC_50_ value of 1.30 ± 0.30 nM. Notably, this is comparable to the 2.42±0.12-fold increase and EC_50_ of 0.68 ± 0.20 nM of latozinemab (**Figures 4B, C**). The difference in EC_50_ values between ABD-A3-PGRN_C_15* and latozinemab was not statistically significant (p=0.19).

## 4 Discussion

Heterozygous loss-of-function mutations in the *GRN* gene lead to FTD through haploinsufficiency (9, 22). Blocking the interaction of PGRN with its clearance receptor sortilin has emerged as a promising therapeutic strategy for FTD-*GRN*, by normalizing the extracellular PGRN levels. To this end, we aimed to develop a high-affinity sortilin-binding affibody-peptide fusion construct, utilizing the fact that PGRN interacts with sortilin through the PGRN C-terminus. Affibody molecules against sortilin were selected by phage display, and later genetically fused with peptides derived from the PGRN C-terminus. For the anti-sortilin affibody A3, fusion with the PGRN C-terminus demonstrated avidity effects and a significantly decreased dissociation rate, indicating that the A3 and PGRN_C_ epitopes are compatible for simultaneous binding to sortilin. Optimization of the peptide moiety of the A3-PGRN_C_ fusion construct, as summarized in **Figure 3**, enabled an up to 380-fold improvement in K_D_ of the fusion construct compared to the parental affibody alone.

In line with previous findings (29), the optimization of the A3-PGRN_C_ fusion construct shows that the very C-terminal-most amino acids of PGRN are sufficient to convey sortilin binding (ABD-A3-PGRN_C_6*), while a slightly longer peptide yields a higher affinity of the fusion construct (e.g. ABD-A3-PGRN_C_15*). Our results, however, indicate that the N-terminal amino acids of these PGRN-derived peptides mainly serve as a spacer, with the PGRN_C_3 peptide harboring the key residues for sortilin binding, as PGRN_C_3 with a flexible linker conveys a similar binding contribution to PGRN_C_6* (ABD-A3-(G_4_S)_3_-PGRN_C_3 *vs*. ABD-A3-(G_4_S)_3_-PGRN_C_6*). This is furthermore in agreement with the A588G mutation not affecting the PGRN affinity for sortilin (33), and indicates that the N-terminal amino acids of the optimal fusion peptide PGRN_C_15* might be amenable to further mutations, for instance to increase stability of the fusion construct *in vivo*.

To investigate the potential of the fusion construct as an FTD-*GRN* therapeutic candidate, the biological activity of A3-PGRN_C_15* was evaluated in a PGRN clearance assay, with latozinemab as comparator. Incubation of sortilin-expressing, PGRN-secreting human glioblastoma U-251 cells with A3-PGRN_C_15* increased the extracellular PGRN levels to the same extent as latozinemab, with an EC_50_ value in the same concentration range. While it should be noted that the biosimilar used in this study may differ from the antibody used in clinical trials, and that the lack of statistically significant difference in EC_50_ values may be an effect of limited statistical power, this nonetheless provides an indication that A3-PGRN_C_15* has an affinity and binding mode suitable for the intended application. It should furthermore be noted that higher affinity of the A3 affibody moiety might be possible to achieve through affinity maturation procedures. Further studies in more biologically relevant FTD models are required to further elucidate the potential of A3-PGRN_C_15* as a therapeutic candidate.

Since their invention, affibody molecules have been investigated extensively for various medical applications [reviewed in (50, 51)]. A therapeutic strategy based on affibody molecules for treatment of FTD-*GRN* would provide an orthogonal approach to the antibody therapies currently under investigation, broadening the spectrum of potential sortilin-targeting therapies. Furthermore, the small size of affibody molecules entails a higher binding site density compared to antibodies, enabling smaller injected volumes and faster administration, likely with similar brain uptake (52–54). In the general case of dementia, and specifically in the case of the genetic FTD-*GRN*, decades-long treatments will most likely be required, making ease of administration and production costs essential factors. The recent approvals of Alzheimer’s disease (AD) antibody drugs aducanumab, lecanemab and donanemab have sparked debate about prohibitive costs in relation to risk/benefit assessments of new dementia therapies. In this context, the bacterial production or chemical synthesis of affibody molecules may provide an attractive option.

The sortilin-binding A3-PGRN_C_ fusion constructs have here been investigated in the context of FTD-*GRN*, but manipulation of the sortilin-PGRN axis might also be of interest in other conditions. There are indications that *GRN* mutations may play a role in other neurodegenerative diseases, such as amyotrophic lateral sclerosis (55), Parkinson’s disease (56), and AD (57–61), with increased PGRN levels having been shown to inhibit plaque formation and protect against amyloid beta toxicity in mouse models of AD (62, 63). Furthermore, there is evidence indicating that blocking the sortilin-neurotensin or sortilin-progranulin interactions might be of interest for the treatment of neuropathic pain (64) and certain forms of breast cancer (65, 66), respectively. Thus, the constructs presented here may have wider applicability than to FTD-*GRN* only.

The present work constitutes the first example of a fusion protein between a peptide and an affibody molecule directed towards the same target, utilizing a combination of directed evolution and naturally occurring receptor ligands to develop high-affinity binders. This approach constitutes an efficient way of greatly increasing the apparent affinity of a binder, and decreasing the need for extensive affinity maturation efforts. While requiring evaluation on a case-by-case basis, the approach could easily be extended to introduce the peptide moiety in the affibody selections. Whereas more established methods of increasing target affinity, such as classic affinity maturation or creation of biparatopic dimeric affibody constructs, have often been successful, the here presented method constitutes an addition to this toolbox of approaches, that may be use in certain cases.

## Data availability statement

The original contributions presented in the study are included in the article/**Supplementary Material**. Further inquiries can be directed to the corresponding author.

## Ethics statement

Ethical approval was not required for the studies on humans in accordance with the local legislation and institutional requirements because only commercially available established cell lines were used.

## Author contributions

ME: Writing – original draft, Validation, Methodology, Investigation, Formal analysis, Data curation, Writing – review & editing, Visualization, Conceptualization. JN: Writing – original draft, Writing – review & editing, Investigation, Formal analysis. P-ÅN: Visualization, Conceptualization, Writing – review & editing. SS: Supervision, Resources, Funding acquisition, Writing – review & editing, Conceptualization. HL: Writing – review & editing, Supervision, Conceptualization. JL: Visualization, Resources, Project administration, Funding acquisition, Writing – review & editing, Supervision, Conceptualization.
