# Supplementary material for: An anti-sortilin affibody-peptide fusion inhibits sortilin-mediated progranulin degradation
Source: Front Immunol. 2024 Aug 8;15:1437886. doi: 10.3389/fimmu.2024.1437886 (PMC11342335; doi:10.3389/fimmu.2024.1437886)
Supplement: Supplementary file 1 [file DataSheet_1.docx]

Supplementary Material

An anti-sortilin affibody-peptide fusion inhibits sortilin-mediated progranulin degradation

**Moira Ek^1^, Johan Nilvebrant^1^, Per-Åke Nygren^1^, Stefan Ståhl^1^, Hanna Lindberg^1^, John Löfblom^1*^**

^1^Department of Protein Science, School of Engineering Sciences in Chemistry, Biotechnology and Health, KTH Royal Institute of Technology, Stockholm, Sweden

**^*^Correspondence:**

John Löfblom: lofblom@kth.se

# Supplementary Figures and Tables

## Supplementary Tables

Table S1. Amino acid sequences of the characterized anti-sortilin affibodies, negative control affibody, ABD035, and human PGRN C*-*terminus carrying the A588G mutation (in bold).

| **Protein moiety** | **Amino acid sequence** |
| --- | --- |
| Negative control affibody Z_wt_ | VDNKFNKEQQNAFYEILHLPNLNEEQRNAFIQSLKD  DPSQSANLLAEAKKLNDAQAPK |
| Anti-sortilin affibody G11 | VDNKFNKENAGARGEIIFLPNLNKNQGWAFKHSLMD  DPSQSANLLAEAKKLNDAQAPK |
| Anti-sortilin affibody F6 | VDNKFNKENVWARGEITFLPNLNSKQHQAFHMSLDD  DPSQSANLLAEAKKLNDAQAPK |
| Anti-sortilin affibody C1 | VDNKFNKERYAAKGEITYLPNLNNKQHMAFDMSLRD  DPSQSANLLAEAKKLNDAQAPK |
| Anti-sortilin affibody A3 | VDNKFNKEIEEAGAEIIQLPNLNRWQKGAFIVSLKD  DPSQSANLLAEAKKLNDAQAPK |
| ABD035 | LAEAKVLANRELDKYGVSDFYKRLINKAKTVEGVE  ALKLHILAALP |
| PGRN_C_21* | LRREAPRWDAPLRDP**G**LRQLL |

## Supplementary Figures

Figure S1. Size exclusion chromatography data for anti-sortilin affibodies. (A)-(D) Elution peaks of Z-ABD format affibodies (A) G11, (B) F6, (C) C1, and (D) A3, in comparison to the archetypal affibody Z_wt_-ABD, demonstrating the typical elution profile of a monomeric affibody-ABD protein. Protein elution was measured by UV absorbance normalized to the main peak within each sample, as a function of elution volume.

Figure S2. Flow cytometric data for binding of control constructs to cancer cells. (A)-(B) The negative control affibody Z_wt_-ABD shows no binding to U-251 (A) or PC-3 (B) cells compared to the secondary agent HSA-Alexa Fluor 647 alone. (C)-(D) Binding of a positive control anti-sortilin antibody confirms the presence of sortilin on U-251 cells (C) but shows very low signals on PC-3 cells (D) compared to secondary antibody only.

Figure S3. Flow cytometric analysis of construct binding to cancer cells. Overview of median fluorescence intensity (MFI) signals normalized to Z_wt_-ABD signal for the respective cell line for all tested ABD035-containing constructs. 100 nM of primary construct was pre-incubated with 200 nM of secondary HSA-Alexa Fluor 647 prior to addition to the cells. The displayed data shows the mean ± SD of n=2 independent experiments.
